# Estimation of the apparent anisotropic water diffusivity on spruce evaluated with a simplified derivative approach and as a function of the flow rate

**DOI:** 10.1038/s41598-026-38932-7

**Published:** 2026-02-07

**Authors:** Antoni Sánchez-Ferrer, Max Engelhardt

**Affiliations:** Wood Materials Science, Wood Research Institute of Munich (HFM), TUM School of Engineering and Design, Technical University of Munich, Winzererstr. 45, 80797 Munich, Germany

**Keywords:** Diffusivity, Dynamic vapor sorption, Fitting models, Anisotropicity, Engineering, Materials science, Mathematics and computing, Physics

## Abstract

The anisotropic apparent diffusivity as a function of the flow rate was determined in a series of Dynamic Vapor Sorption (DVS) experiments on spruce samples in the three orthotropic directions. Four different fitting procedures were applied for the evaluation of the time-sorption isotherms, *i.e.*, the double-stretched exponential (DSE), the Ritger-Peppas (RP), and the Fickian and double-Fickian (SUM/DSUM) fitting methods, together with a derivative (DER) method for the determination of the apparent diffusivity. The results confirm that the DER method delivers similar results to the DSE fitting procedure, *i.e.*, 1.98·10^− 10^, 0.94·10^− 10^ and 1.00·10^− 10^ m^2^/s for the longitudinal, radial and tangential direction, respectively, with a maximum of 10% deviation, in a much simpler manner.

**Supplementary Information:**

The online version contains supplementary material available at 10.1038/s41598-026-38932-7.

## Introduction

The liquid water diffusivity of wood samples can be calculated from the mass uptake as a function of time when imposing a sudden change in the relative humidity of the chamber in a Dynamic Vapor Sorption (DVS) equipment. This setup allows for a continuous logging of the mass sample while controlling temperature, relative humidity and flow rate. The data obtained can be evaluated using several fitting functions from the past, *e.g.*, Higuchi^1^, Ritger-Peppas^2^ and Weibull equations^3^, which are related to the solution of Fick’s law or Crank’s series of exponential functions^4^.

The power-law Higuchi equation is a special case of the Ritger-Peppas function, Δm/Δm_∞_ = [(t-t_0_)/τ]^n^, when only Fickian diffusion processes exist, indicated by a fixed exponent value of *n* = 0.5, while non-Fickian diffusion cases (*n* > 0.5) are more precisely described by the Ritger-Peppas (RP) function^5^. But, both power-law equations can only be applied to the data covering the initial 40% of the total amplitude of the data, omitting the rest at longer times^6,7^. Moreover, both are simplified solutions to the Crank’s series of exponential functions (SUM).

The Crank and Weibull equations are related to exponential decay equations, where the former is a series of such a function^4^ and the latter is equivalent to a distribution of exponential functions summarized into the so-called single-stretched exponential function (SSE)^8^ or equivalent to a Prony series with a finite number of terms^9^. The Weibull equation explains well processes related to the fractional kinetic model^10^, *e.g.*, sorption at the solid/solution interface^11^, fractal reaction kinetics^12^, stochastic theory of relaxation^13^, and arbitrary-order reaction kinetics^14^. To close the circle, the Weibull function can be rewritten by taking a Taylor series expansion, with a resulting expression similar to that of RP for short times^6,15^.

When evaluating the sorption results from natural^16^ and synthetic materials^17^, the data could not be fitted adequately by any of these previously mentioned models, *i.e.*, RP, SUM, or SSE, and a dual-stage Crank’s series (DSUM) model consisting of two Fickian diffusion kinetics^4^ occurring in parallel was implemented. Alternatively, a new mathematical approach, the double-stretched exponential (DSE) function or double-Weibull function, was introduced^7^ that could better explain the experimental data obtained from DVS experiments for the analysis of wood samples^18–20^.

Several works have already mentioned the two-diffusion behavior in wood. Due to a higher porosity in the cell wall compared to the middle lamella, molecular tracer diffused faster in the former than in the latter. The cell wall contains *ca.* 20% of lignin, whereas the middle lamella contains *ca.* 80%, making the middle lamella less porous compared to the cell wall, reducing water mobility and, therefore, lowering the diffusivity in this highly crosslinked and amorphous lignin-rich domain^21^. In the same spirit, ion diffusion happened to be correlated with the segmental polymer motion, which is lower for the crosslinked lignin middle lamella domain, proving that ion conductivity in the cell wall was, at least, 1 order of magnitude higher than in the middle lamella^22^. These last results were in line with the electrical resistance experiments performed in an earlier work^23^. With all this information and accepting that both the cell wall and the middle lamella are combinations of different compositions and morphologies, the use of two parallel diffusion curves based on the double-stretched exponential (DSE) function has to be considered.

In a previous communication, the apparent diffusion coefficient D_app_ was correlated to the flow rate Q when obtaining the moisture sorption isotherm on a disk sample in the L-direction^24^. The results showed how crucial it is to work at high Q values in order to diminish the concentration gradient toward the surface of the sample. The established procedures for the D_app_ calculation start with fitting the experimental data and calculating the corresponding lifetime value τ, followed by imposing the shape factor F of the sample to obtain the corresponding diffusivity: D_app_ = F/τ. That is a tedious process, which is affected by the expertise and skills of the scientist who analyzes the data, and for all these above-described methods, long measurements and complex data-fitting algorithms are required.

In this paper, we show a simple method for the evaluation of the diffusion coefficient based on a simple mathematical approach based on the Gumbel density function^25,26^ – determination of the peak maxima in a derivative curve of the experimental data – that was implemented during the evaluation of thin spruce disk specimens sealed at their curved face in the three orthotropic directions during adsorption experiments – from 30% to 80% RH – performed in a DVS equipment at different gas flow conditions. Moreover, we compare the results to the ones obtained when using the DSE, the power-law Ritger-Peppas (RP), the Fickian (SUM) and the double-Fickian (DSUM) series fitting functions in terms of flow constant k and maximum apparent diffusivity values $\:{\mathrm{D}}_{\mathrm{a}\mathrm{p}\mathrm{p}}^{\mathrm{m}\mathrm{a}\mathrm{x}}$ in the three directions.

## Materials and methods

### Materials

Spruce disk samples of 12.0 mm in diameter and *ca.* 1.0 mm in thickness were cut in the L-, R- and T-directions with an equilibrium mass (m_0_) at 30% RH of 44.1, 46.6 and 42.1 mg, respectively. The lateral surface of the disks was coated with a hydrophobic acrylic coating under the microscope, resulting in a final mass (m_disk_ = m_0_ + m_coating_) at 30% RH of 54.5, 62.1 and 53.0 mg for the disks in the L-, R- and T-directions, respectively.

## Dynamic vapor sorption (DVS) experiments

Dynamic Vapor Sorption (DVS) experiments were conducted using a gravimetric vapor sorption device (DVS Advantage ET, Surface Measurement Systems). The device is equipped with a microbalance of 0.1 µg sensitivity and a chamber that is purged with a nitrogen flow Q of 10 to 200 cm^3^/min (0.6 to 12 L/h) at a selected RH value obtained by mixing different flows of dry and water-saturated nitrogen.

Samples were measured by attaching two ring-shaped aluminum sample holders to the electronic beam balance of the DVS. The sample was placed in one of them while the other was used as an empty reference to eliminate sorption effects from the holders.

After conditioning the sample at 25 °C and 30% RH for *ca.* 2 days to achieve mass equilibrium, the measurement started by changing the RH of the gas flow to 80% RH and monitoring the sample mass gain for a total of 48 h.

## Methods for the determination of the apparent diffusion coefficient

The apparent diffusion coefficient D_app_^27^ was calculated using the lifetime value τ and thickness of the samples $\:l$ as follows (Eq. 1):

1 $${D}_{app}=\frac{\pi\:}{16}\frac{{l}^{2}}{\tau\:}$$

In order to minimize the effect due to the evolution of the RH in the DVS chamber when ramping from 30% to 80% RH, the sorption data used for the evaluation started when the RH in the DVS chamber reached 95% of the RH step (0.95 ΔRH), *i.e.*, ideally 77.5% RH in this work, as introduced in Appendix SI-A ^7^.

All dynamic moisture adsorption steps were analyzed using a double-stretched exponential (DSE) model (Eq. 2) ^7^, to obtain an equivalent lifetime value τ.

2 $$\frac{\varDelta\:m}{\varDelta\:{m}_{\infty\:}}=1-\sum\limits_{i=1}^{2}{A}_{i}{e}^{-{\left(\frac{t-{t}_{0}}{{\tau\:}_{i}}\right)}^{{\beta\:}_{i}}}$$

where Δm/Δm_∞_ = [m(t)-m_0_]/[m_∞_–m_0_] is the normalized mass uptake, A is the amplitude, τ is the lifetime, and β is the stretched exponential factor or shape factor.

The power-law Ritger-Peppas (RP) (Eq. 3), the Fickian series (SUM) (Eq. 4) and the double-Fickian series (DSUM) (Eq. 5) models were also used for comparison.

3 $$\frac{\varDelta\:m}{\varDelta\:{m}_{\infty\:}}=A{\left(\frac{t-{t}_{0}}{\tau\:}\right)}^{n}$$

4 $$\frac{\varDelta\:m}{\varDelta\:{m}_{\infty\:}}=A\left[1-\sum\limits_{i=0}^{\infty\:}\frac{8}{{\left(2i+1\right)}^{2}{\pi\:}^{2}}{e}^{-\frac{{\left(2i+1\right)}^{2}{\pi\:}^{2}D\left(t-{t}_{0}\right)}{{l}^{2}}}\right]$$

5 $$\frac{\varDelta\:m}{\varDelta\:{m}_{\infty\:}}=\sum\limits_{i=1}^{2}{A}_{i}\left[1-\sum\limits_{j=0}^{\infty\:}\frac{8}{{\left(2j+1\right)}^{2}{\pi\:}^{2}}{e}^{-\frac{{\left(2j+1\right)}^{2}{\pi\:}^{2}{D}_{i}\left(t-{t}_{0}\right)}{{l}^{2}}}\right]$$

where n is the Ritger-Peppas exponent, $\:D$ is the Fickian diffusion coefficient, and $\:l$ is the thickness of the sample. The lifetime value τ can be defined as the time needed to achieve 63.2% of the total sorption process or when Δm/Δm_∞_ = 0.632 for a single exponential process. The corresponding D_0.63_ can be calculated using Eq. (1)

In addition to all these models, a simplified method based on the half-life time of the sorption process t_0.5_ when Δm/Δm_∞_ = 0.5^28^ has also been applied to derive the diffusion coefficient (Eq. 6).

6 $${D}_{0.5}=-\frac{1}{{\pi\:}^{2}}ln\left[\frac{{\pi\:}^{2}}{16}-\frac{1}{9}{\left(\frac{{\pi\:}^{2}}{16}\right)}^{9}\right]\frac{{l}^{2}}{{t}_{0.5}}=0.049\frac{{l}^{2}}{{t}_{0.5}}$$

## Derivative method for the determination of the apparent diffusion coefficient

**Fig. 1:**
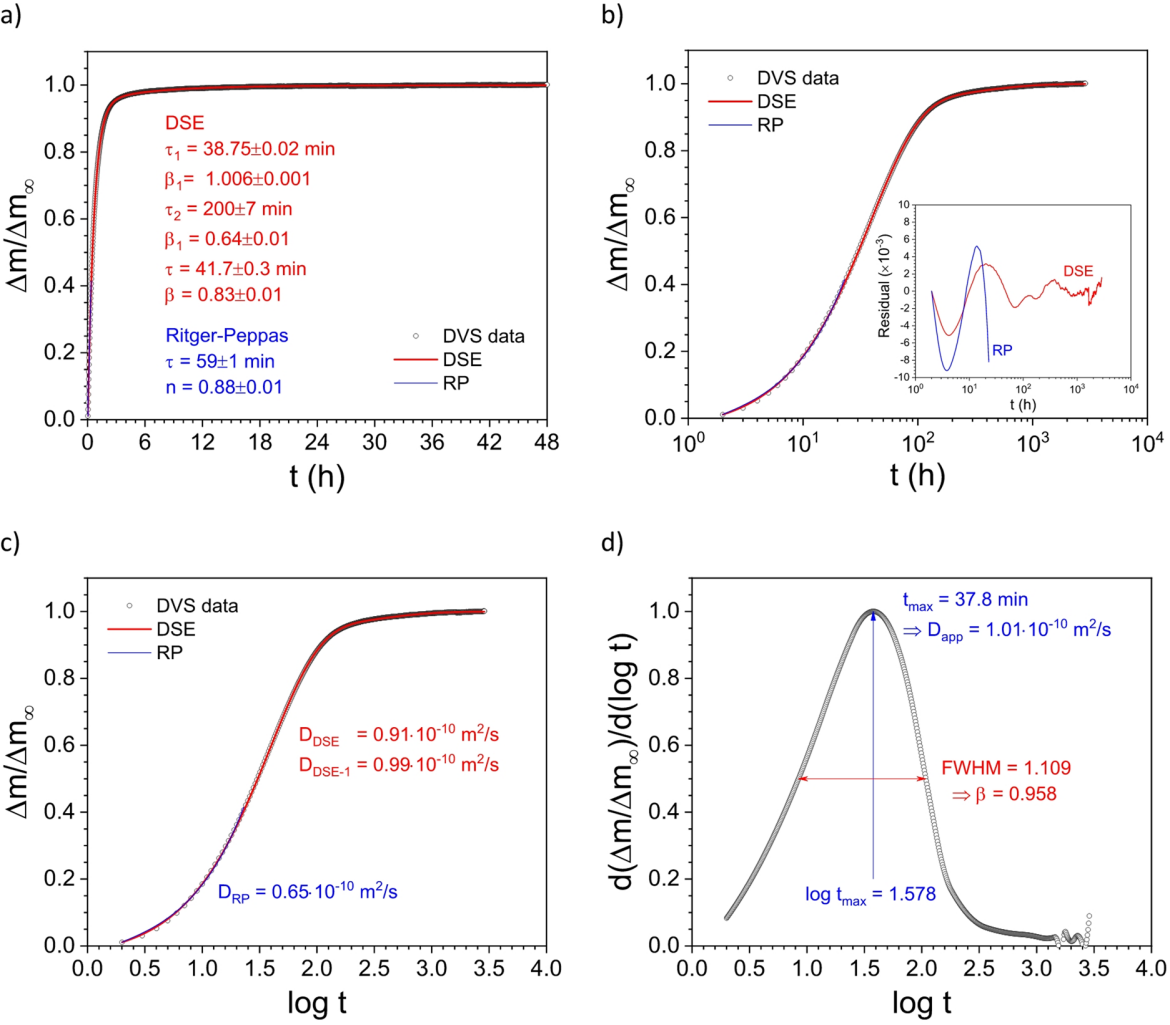
(**a**) lin-lin and (**b**) lin-log plot of the time-sorption isotherm DVS data together with the DSE (red) and the RP (blue) fitting curves. (**c**) lin-log plot of the time-sorption isotherm DVS data with the abscissa values as log t, together with the DSE (red) and the RP (blue) fitting curves. (**d**) lin-log plot of the time-sorption isotherm DVS data derivative with the corresponding peak maximum (log t_max_) and the full width at half maximum (FWHM).

The derivative method (DER) allows for the determination of the apparent diffusion coefficient by evaluating the time value at the local maximum t_max_ in the derivative curve of the Δm/Δm_∞_ *vs.* log t. The procedure consists of a few simple mathematical steps:

1. Normalize the time-sorption isotherm DVS data: y = Δm/Δm∞ = [m(t)-m0]/[m∞-m0] (Fig. 1A,B).
2. Calculate the logarithm of all t values, *i.e.*, x = log t, and plot y *vs.* x, *i.e.*, Δm/Δm∞ *vs.* log t (Fig. 1C).
3. Calculate the corresponding derivative dy/dx, *i.e.*, d(Δm/Δm∞)/d(log t), and plot dy/dx *vs.* x, *i.e.*, d(Δm/Δm∞)/d(log t) *vs.* log t (Fig. 1D).
4. Localize the x value corresponding to the maximum in the curve dy/dx *vs.* x, *i.e.*, xmax = log t_max_, where t_max_ = $10^{{x_{{\max }} }}$
5. Calculate the apparent diffusion coefficient by applying Eq. 1, *i.e.*, substitute τ with the obtained t_max_ value.

Moreover, the full width at half maximum (FWHM) of the derivative curve in Fig. 1D allows for estimating the shape factor β of the curve. This shape factor correlates with the shape factor β_1_ of the fast sorption process from the DSE method.

More details regarding the DER method can be found in Appendix SI-A, *e.g.*, the detection of the peak maximum t_max_ for the lifetime τ calculation with the corresponding apparent diffusion parameter D_app_, and evaluation of the peak’s FWHM for the calculation of the shape factor β. Moreover, a detailed explanation about the Gumbel density function for the analysis of the peak derivative can be found in Appendix SI-B.

## Results and discussion

In order to observe the effect of the flow rate Q for the three orthotropic wood directions, a single adsorption isotherm step from 30% to 80% RH was conducted at different Q values. By fitting the mass gain with the double-stretched exponential (DSE) function ^7^, the lifetime values τ_1_ and τ_2_, which correspond to the fast and slow adsorption processes, respectively, were determined (Fig. SI-1, SI-2 and SI-3). The corresponding equivalent lifetime τ value was calculated by applying a minimization process (Appendix SI-A: ^7^, and the apparent diffusion coefficient D_DSE_ was calculated for each Q value and for the three wood directions (Table SI-1, SI-2 and SI-3).

The apparent diffusion coefficient values D_RP_, D_SUM_ and D_DSUM_ following the Ritger-Peppas (RP) (Fig. SI-4, SI-5, SI-6), the Fickian (SUM) (Fig. SI-7, SI-8, SI-9) and the double-Fickian (DSUM) (Fig. SI-10, SI-11, SI-12) models, respectively, were also calculated (TableSI-4, SI-5, SI-6, SI-7 and SI-8). Finally, to validate all models, the apparent diffusion coefficient from the lifetime value D_0.63_ and from the half-life time value D_0.5_ were determined (Table SI-9 and SI-10).

Parallelly, the derivative (DER) method was implemented. From the exponential growth of the adsorption processes, the maximum slope of the S-shape curve - when plotting the relative mass Δm/Δm_∞_ = [m(t)–m_0_]/[m_∞_–m_0_] *vs.* log t - was determined by localizing the local maximum in the corresponding first derivative curve. The abscissa at this peak maximum t_max_ is close to the lifetime τ_1_ of the fast sorption process obtained from the DSE approach, contributing to more than 90% of the total diffusion at high Q values. The apparent diffusion coefficient D_DER_ (Table SI-11) and the corresponding shape factor β (Table SI-12) were calculated from the t_max_ and the FWHM of the peak maxima, respectively. A complete step-by-step description of the derivative (DER) method, together with some plots detailing the procedure, can be found in Appendix SI-A.

**Fig. 2:**
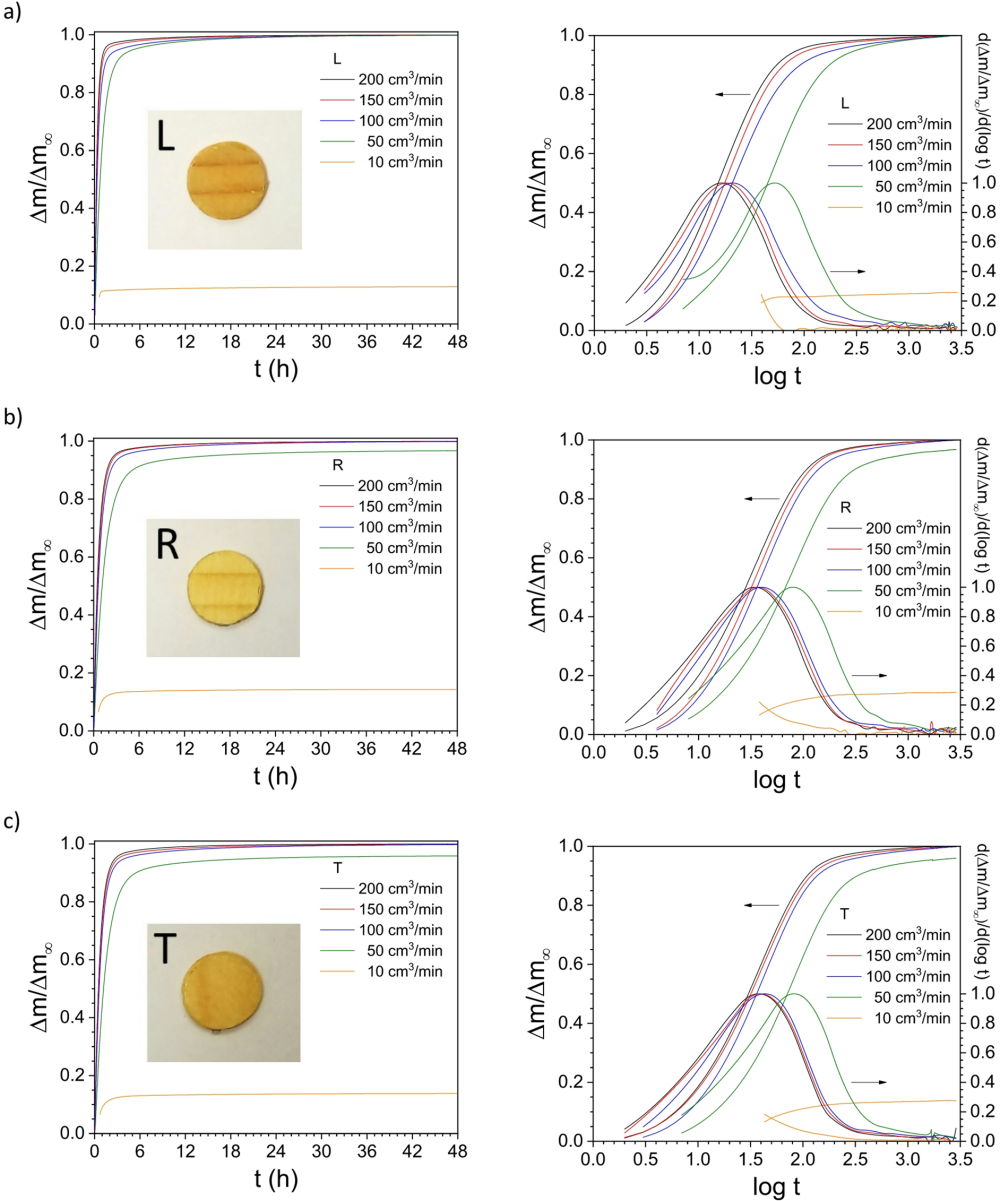
Normalized time-sorption curves from 30% to 80% RH at different flow rates Q,* i.e.*, 200, 150, 100, 50 and 10 cm^3^/min, for the three wood directions: (**a**) longitudinal (L), (**b**) radial (R), and (**c**) tangential (T). The plots on the left column are the lin-lin curves, while the plots on the right are lin-log curves. The plots on the right also include the corresponding normalized derivatives (peak function) of the sigmoidal curves. *Note* the time-sorption isothermal data were normalized with respect to the maximum water uptake obtained at 200 cm^3^/min.

In Fig. 2, the normalized time-sorption curves - or the relative time-mass Δm/Δm_∞_ evolution - at different Q values for the three orthotropic directions (Fig. 2, left column), and the lin-log time-sorption curves and the corresponding derivative for each curve (Fig. 2, right column) are shown. From the lin-lin plots (Fig. 2, left column), a slowdown process is already noticeable when the flow rate decreases. Moreover, the diffusivity in the L-direction appears to be faster than that in the R- and T-directions. Exceptionally, at Q = 10 cm^3^/min, the sample reached an equilibrium with a moisture content lower than the *ca.* 8% increase corresponding to the step from 30 to 80% RH. This lower value in the equilibrium moisture content is due to the small number of water molecules available to interact with the samples’ surface at low flow rates^29^, even though the water concentration or RH value was the same for all studied flow rates.

The speed of the process is better visualized in the S-shape lin-log plots (Fig. 2, right column). The corresponding derivatives clearly indicate a shift in the local maximum upon decreasing the flow rate value, which points out a slowing down of the diffusion process due to fewer available water molecules close to the wood surfaces in the viscous gas sublayer^30^. For samples under the flow rate value Q = 50 cm^3^/min, thus, it was impossible to evaluate and detect the maximum in the derivative.

The fast and the equivalent lifetime values τ_1_ and τ, respectively, from the DSE fitting analysis at different flow rate values – Q = 50, 100, 150 and 200 cm^3^/min – and for the L-, R- and T-directions are summarized in Table SI-1, SI-2 and SI-3, respectively. The time corresponding to the peak maximum t_max_ in the derivative curves (Fig. 2, right column) at different flow rate values – Q = 50, 100, 150 and 200 cm^3^/min – for the three wood directions are collected in Table SI-11, and the values are remarkably quite similar to those of the fast τ_1_ values from the DSE fitting approach. Finally, the lifetime values τ from the RP fitting analysis at different flow rate values – Q = 50, 100, 150 and 200 cm^3^/min – for the three wood directions are summarized in Table SI-4, which strongly deviate from the DSE and DER methods and shows that the widely used power-law RP method is not suitable for the water diffusivity analysis in wood.

**Fig. 3:**
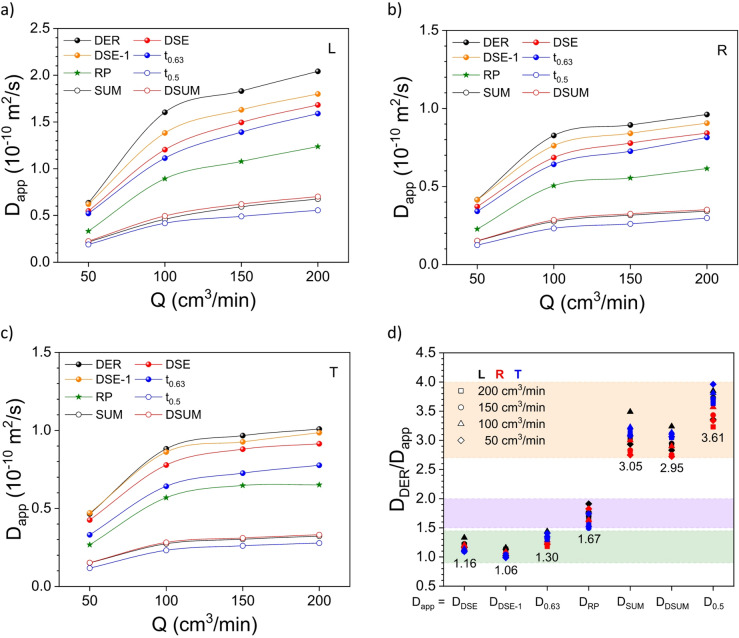
Apparent diffusion coefficient D_app_ as a function of the flow rate Q for the three wood directions, *i.e.*, (**a**) L, (**b**) R, and (**c**) T, obtained from the different methods, *i.e.*, derivative (DER; black filled circle), double-stretched exponential (DSE; red filled circle – fast diffusion process DSE-1; orange filled circle), lifetime (t_0.63_; blue filled circle), Ritger-Peppas (RP; green star), Fickian series (SUM; black empty circle), double-Fickian series (DSUM; red empty circle), and half-life time (t_0.5_; blue empty circle). (**d**) Relative apparent diffusion coefficient between the values obtained from the DER method compared to the other methods for the different flow rate values and wood directions. *Note*: the number below each group is the average value.

When comparing the apparent diffusion coefficient values calculated from the different methods, *i.e.*, DSE, RP, SUM, DSUM and DER methods, together with the values obtained from the lifetime t_0.63_ and the half-life time t_0.5_ approach, all methods can be grouped (Fig. 3). The first group includes the apparent diffusion coefficient values from the DER and DSE methods and from the lifetime calculation, whose relative values are between 1.0 and 1.45. The second group comprises the apparent diffusion coefficient values from the SUM and DSUM methods and from the half-life time calculation, whose relative values are between 2.7 and 4.0. And finally, there are the apparent diffusion coefficient values from the RP approach, whose relative values are between 1.5 and 2.0. This analysis clearly indicates that the DER method results are very similar to those obtained from the DSE approach, which accounts for all experimental points along the 48 h experiment with a minimum residue compared to the other methods.

**Fig. 4:**
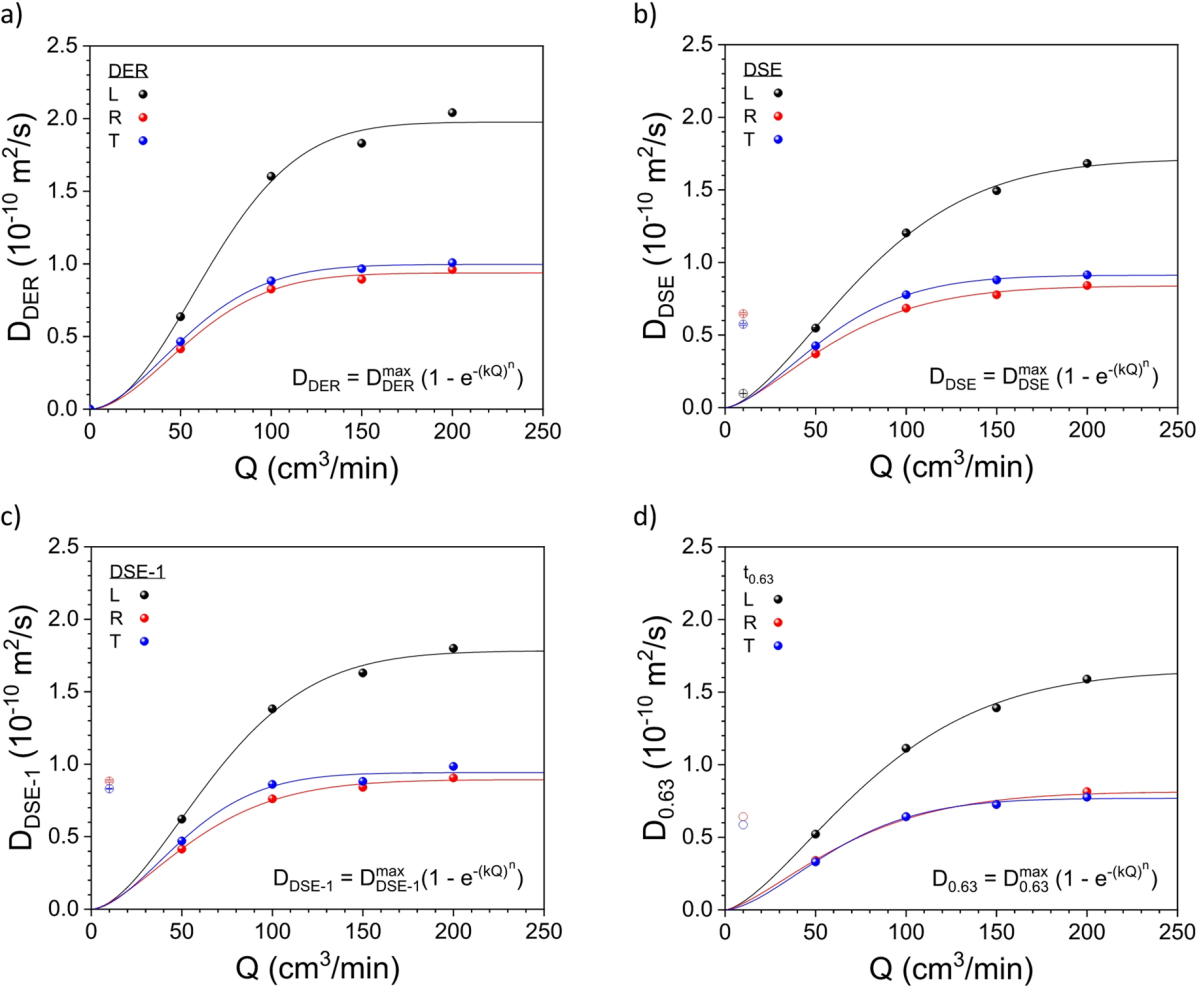
Apparent diffusion coefficient (D_app_) values from 30 to 80% RH as a function of the flow rate (Q) for the three wood directions,* i.e.*, L, R and T, obtained from (**a**) the t_max_ values in Fig. 1 using the DER method (right column plots), (**b**) the fitting of the time-sorption isotherms in Fig. 1 using the DSE function, (**c**) the fast sorption process from the fitting of the time-sorption isotherms in Fig. 1 using the DSE function, and (**d**) the lifetime (t_0.63_) of the time-sorption isotherms in Fig. 1. The curves show the corresponding fitting results to the data in the three wood directions following a stretched exponential function.

Because the results obtained from the DER method and the fast diffusion component from the DSE method were quite similar, the apparent diffusion coefficient values from the former, D_DER_, were correlated to the values from the latter, D_DSE−1_. The results show a good linear correlation between both data sets: D_DSE−1_ = 0.91 D_DER_ (R^2^ = 0.998). Similarly, a linear correlation was found between the shape factor from both data sets: β_1_ = 0.996 β (R^2^ = 0.9990). In Fig. SI-13, both linear correlations are shown, indicating that the DER method allows for determining τ, β and D_app_ values with similar results to those of the DSE model but in an easier way. From the analysis of the shape factor β, *i.e.*, 0.98 < β_L_ < 1.09, 0.98 < β_R_ < 1.05, and 1.01 < β_T_ < 1.10, we can conclude that the moisture transport phenomenon in spruce can be classified as a non-Fickian diffusion process (0.59 < β < 1.15 from the DSE method or 0.5 < *n* < 1 from the RP method) (Online Appendix SI-A: ^7^,.

The apparent diffusion coefficient values were correlated to the corresponding Q value for the three wood directions following a concave exponential function $\:{\mathrm{D}}_{\mathrm{a}\mathrm{p}\mathrm{p}}={\mathrm{D}}_{\mathrm{a}\mathrm{p}\mathrm{p}}^{\mathrm{m}\mathrm{a}\mathrm{x}}\left(1-{\mathrm{e}}^{-{\left(\mathrm{k}\mathrm{Q}\right)}^{n}}\right)$^24^. Figure 4 shows this correlation for the apparent diffusion coefficient values obtained using the methods in the first group, *i.e.*, the DER and DSE methods and the lifetime calculation. Since the model fails at low Q values, the D_app_ values at Q = 10 cm^3^/min were excluded in all fitting procedures (Fig. 4 empty symbols). The same procedure was also applied for the apparent diffusion coefficient values from the different methods, *i.e.*, RP, SUM, DSUM and half-life time. All fitting plots for the different methods are presented in Fig. SI-14 (for *n* = 1, a CDF for an exponential distribution; and for *n* > 1, a stretched exponential distribution). The flow rate constant k and the maximum apparent diffusion coefficient (when Q → ∞) for the different studied methods can be found in Table SI-13 and SI-14.

**Fig. 5:**
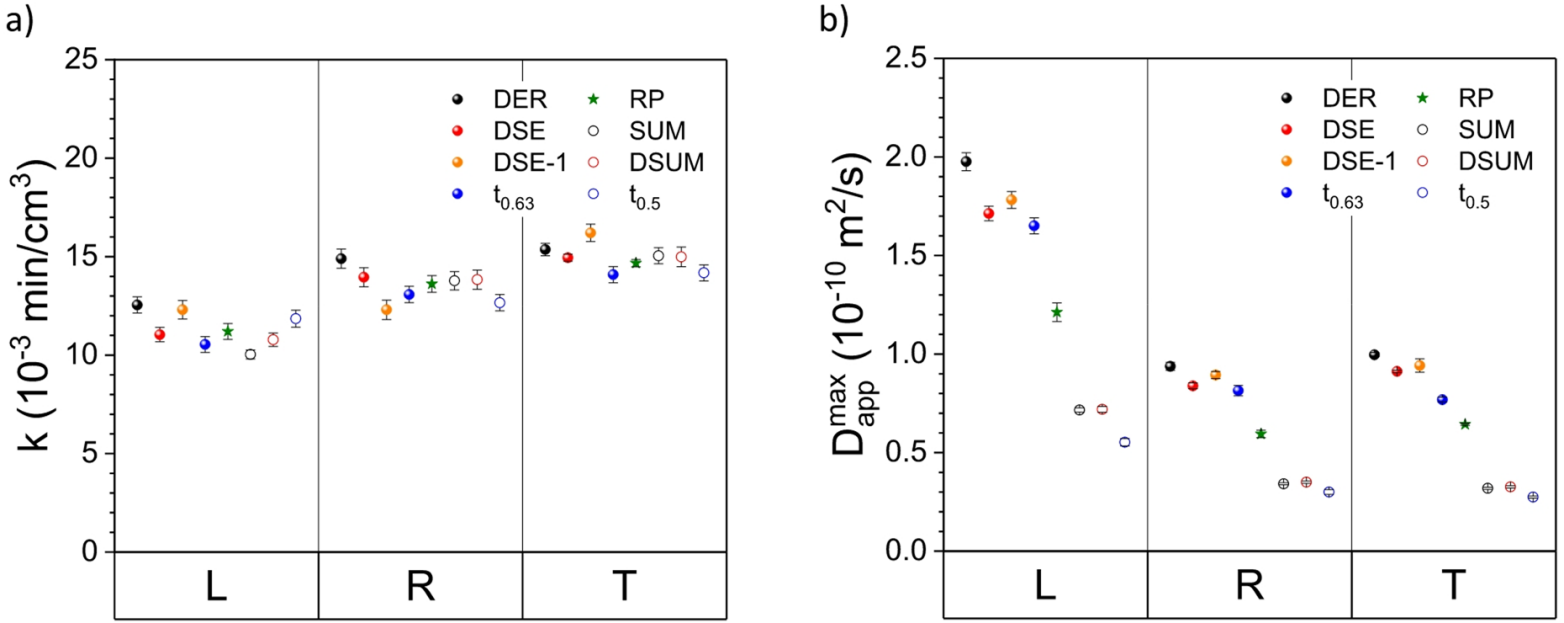
(**a**) Flow rate constant (k) and (**b**) maximum apparent diffusion coefficient ($\:{\mathrm{D}}_{\mathrm{a}\mathrm{p}\mathrm{p}}^{\mathrm{m}\mathrm{a}\mathrm{x}}$) from 30 to 80% RH as a function of the flow rate (Q) for the three wood directions, *i.e.*, L, R and T, obtained from the fits in Figs. 4 and SI-14.

The flow rate constant k for the first group of methods, *i.e.*, DER, DSE and lifetime, and for the L-, R- and T-direction ranges from 10.5 to 12.5 × 10^− 3^ min/cm^3^, from 12.3 to 14.9 × 10^− 3^ min/cm^3^, and from 14.1 to 16.2 × 10^− 3^ min/cm^3^, respectively (Fig. 5a). Moreover, the maximum apparent diffusivity $\:{\mathrm{D}}_{\mathrm{a}\mathrm{p}\mathrm{p}}^{\mathrm{m}\mathrm{a}\mathrm{x}}$ for the same group of methods ranges from 1.65 to 1.98 × 10^− 10^ m^2^/s, from 0.81 to 0.94 × 10^− 10^ m^2^/s, and from 0.77 to 1.00 × 10^− 10^ m^2^/s, for the L-, R- and T-directions, respectively, and are quite similar when comparing the values from the DSE and DER data sets – maximum of *ca.* 10% deviation (Fig. 5b) – and those from early works^7,19,24^. Contrary to that, the maximum apparent diffusivity values from the RP and the SUM/DSUM analysis resulted in values *ca.* 1.6 and 3 times lower than those from the DER approach.

## Conclusions

This study demonstrated that the derivative (DER) approach for the determination of the lifetime τ and, therefore, the apparent water diffusivity D_app_ in wood is a simple procedure that delivers comparable values to those from the fitting procedure of the data, with a maximum deviation in relative differences of 10%. Spruce disk samples in the three orthotropic directions were measured in a DVS equipment at different flow rate values and evaluated with the double-stretched exponential (DSE) fitting method, among others, *i.e.*, Ritger-Peppas (RP) and Fickian (SUM/DSUM) methods, together and the derivative (DER) approach. From these results, the flow rate dependency of the apparent diffusivity was found to follow a concave exponential function, and the flow rate constant k and the maximum apparent diffusivity $\:{\mathrm{D}}_{\mathrm{a}\mathrm{p}\mathrm{p}}^{\mathrm{m}\mathrm{a}\mathrm{x}}$ in the three orthotropic wood directions were calculated. Finally, the results were compared to the commonly used power-law RP and Crack’s series SUM/DSUM fitting approaches, with values for those two models of 1.5 and 3 times, respectively, lower than the apparent diffusivity values from the DER method. Therefore, the DER approach was found to provide a simple and, at the same time, reliable method that avoids long measurements (in this series, t_max_ was reached in well under 90 min) and complex data-fitting algorithms.

## Supplementary Information

Below is the link to the electronic supplementary material.

Supplementary Material 1

## Data Availability

The authors declare that the data supporting the findings of this study are available within the paper and its Supplementary Information files. Should any raw data files be needed in another format, they are available from the corresponding author upon reasonable request.

## References

[1] Higuchi, T. Rate of release of medicaments from ointment bases containing drugs in suspension. *J. Pharm. Sci.* **50**, 874–875. 10.1002/jps.2600501018 (1961). 13907269 10.1002/jps.2600501018

[2] Ritger, P. L. & Peppas, N. A. A simple equation for description of solute release I. Fickian and non-Fickian release from non-swellable devices in the form of slabs, spheres, cylinders or discs. *J. Control Release*. **5**, 23–36. 10.1016/0168-3659(87)90034-4 (1987). 25356469

[3] Weibull, W. A statistical distribution function of wide applicability. *J. Appl. Mech.* **18**, 293–297. 10.1115/1.4010337 (1951).

[4] Crank, J. *The Mathematics of Diffusion* 2nd edn (Clarendon, 1975).

[5] Siepmann, J. & Peppas, N. A. Higuchi equation: derivation, applications, use and misuse. *Int. J. Pharm.* **418**, 6–12. 10.1016/j.ijpharm.2011.03.051 (2011). 21458553 10.1016/j.ijpharm.2011.03.051

[6] Negrini, N., Sánchez-Ferrer, A. & Mezzenga, R. Influence of electrostatic interactions on the release of charged molecules from lipid cubic phases. *Langmuir* **30**, 4280–4288. 10.1021/la5008439 (2014). 24673189 10.1021/la5008439

[7] Sánchez-Ferrer, A., Engelhardt, M. & Richter, K. Anisotropic Wood–Water interactions determined by gravimetric vapor sorption experiments. *Cellulose* **30**, 3869–3885. 10.1007/s10570-023-05093-z (2023).

[8] Zeng, Q. & Xu, S. A two-parameter stretched exponential function for dynamic water vapor sorption of cement-based porous materials. *Mater. Struct.* **50**, 128. 10.1617/s11527-017-0997-7 (2017).

[9] Mauro, J. C. & Mauro, Y. Z. On the prony series representation of stretched exponential relaxation. *Phys. A: Stat. Mech. Appl.* **506**, 75–87. 10.1016/j.physa.2018.04.047 (2018).

[10] Brouers, F. & Sotolongo-Costa, O. Generalized fractal kinetics in complex systems (application to biophysics and biotechnology). *Phys. A*. **368**, 165–175. 10.1016/j.physa.2005.12.062 (2006).

[11] Haerifar, M. & Azizian, S. Fractal-like adsorption kinetics at the solid/solution interface. *J. Phys. Chem. C*. **116**, 13111–13119. 10.1021/jp301261h (2012).

[12] Kopelman, R. Fractal reaction kinetics. *Science* **241**, 1620–1626. 10.1126/science.241.4873.1620 (1988). 17820893 10.1126/science.241.4873.1620

[13] Jurlewicz, A. & Weron, K. A general probabilistic approach to the universal relaxation response of complex systems. *Cell. Mol. Biol. Lett.* **4**, 55–86 (1999).

[14] Niven, R. K. q-Exponential structure of arbitrary-order reaction kinetics. *Chem. Eng. Sci.* **61**, 3785–3790. 10.1016/j.ces.2005.12.004 (2006).

[15] Corsaro, C., Neri, G., Mezzasalma, A. M. & Fazio, E. Weibull modeling of controlled drug release from Ag-PMA nanosystems. *Polymers* **13**, 2897. 10.3390/polym13172897 (2021). 34502937 10.3390/polym13172897 PMC8434431

[16] Célino, A., Fréour, S., Jacquemin, F. & Casari, P. Characterization and modeling of the moisture diffusion behavior of natural fibers. *J. Appl. Polym. Sci.* **130**, 297–306. 10.1002/app.39148 (2013).

[17] Loh, W. K., Crocombe, A. D., Abdel Wahab, M. M. & Ashcroft, I. A. Modelling anomalous moisture uptake, swelling and thermal characteristics of a rubber toughened epoxy adhesive. *Int. J. Adhes. Adhes.* **25**, 1–12. 10.1016/j.ijadhadh.2004.02.002 (2005).

[18] Huang, L., Tang, Y., Liu, W., Hu, Q. & Wei, X. Cellulose paper-based humidity power generator with high open circuit voltage based on zinc-air battery structure. *Carbohydr. Polym.* **326**, 121649. 10.1016/j.carbpol.2023.121649 (2024). 38142083 10.1016/j.carbpol.2023.121649

[19] Wicher, A., Swirska-Perkowska, J. & Pochwała, S. Influence of humidity fluctuations in the chamber on the calculated values of the moisture diffusion coefficient in wood. *Arch. Civ. Eng.* **70**, 205–224. 10.24425/ace.2024.150979 (2024).

[20] Yin, F. et al. Moisture sorption and its induced deformation of juvenile wood at different radial positions in Poplar wood at the tissue scale. *Ind. Crops Prod.* **220**, 119435. 10.1016/j.indcrop.2024.119435 (2024).

[21] Donaldson, L. A., Cairns, M. & Hill, S. J. Comparison of micropore distribution in cell walls of softwood and hardwood xylem. *Plant. Physiol.* **178**, 1142–1153. 10.1104/pp.18.00883 (2018). 30217826 10.1104/pp.18.00883 PMC6236611

[22] Jakes, J E. Mechanism for diffusion through secondary cell walls in lignocellulosic biomass. *J. Phys. Chem. B*. **123**, 4333–4339 (2019). 10.1021/acs.jpcb.9b01430. 31020839 10.1021/acs.jpcb.9b01430

[23] Zelinka, S. L. et al. Cell wall domain and moisture content influence Southern pine electrical conductivity. *Wood Fiber Sci.* **48**, 54–61 (2016). https://wfs.swst.org/index.php/wfs/article/view/2339

[24] Sánchez-Ferrer, A. & Engelhardt, M. Determination of the water diffusivity dependence with the flow rate using a DVS equipment. (2025). 10.1007/s00107-024-02182-z

[25] Gumbel, E. J. Les valeurs extrêmes des distributions statistiques. *Ann. De l’Institut Henri Poincaré*. **5**, 115–158 (1935). http://archive.numdam.org/article/AIHP_1935__5_2_115_0.pdf

[26] Johnson, N., Kotz, S. & Balakrishnan, N. *Continuous Univariate Distributions* 2nd edn (Wiley, 1995).

[27] Neogi, P. *Diffusion in Polymers* (Marcel Dekker, 1996).

[28] Mossner, L. S. Design of sorption experiments for concentrated polymer solutions above T_g_. *Mich. State Univ.*10.25335/b7m6-kv84 (1986).

[29] Söderström, O. & Salin, J. G. On determination of surface emission factors in wood drying. *Holzforschung* **47**, 391–397. 10.1515/hfsg.1993.47.5.391 (1993).

[30] Schlichting, H. & Gersten, K. *Boundary-Layer Theory* (Springer, 2017). 10.1007/978-3-662-52919-5

