# Supplementary material for: Estimation of the apparent anisotropic water diffusivity on spruce evaluated with a simplified derivative approach and as a function of the flow rate
Source: Sci Rep. 2026 Feb 7;16:5876. doi: 10.1038/s41598-026-38932-7 (PMC12894689; doi:10.1038/s41598-026-38932-7)
Supplement: Supplementary file 1 — Supplementary Material 1 [file 41598_2026_38932_MOESM1_ESM.docx]

SUPPORTING INFORMATION

**Estimation of the Apparent Anisotropic Water Diffusivity on Spruce Evaluated with a Simplified Derivative Approach and as a Function of the Flow Rate**

Antoni Sánchez-Ferrer^1,^*, Max Engelhardt^1^

^1^ Wood Materials Science, Wood Research Institute of Munich (HFM), TUM School of Engineering and Design, Technical University of Munich, Winzererstr. 45, 80797 Munich, Germany

Antoni Sánchez-Ferrer*: orcid.org/0000-0002-1041-0324; email: [sanchez@hfm.tum.de](mailto:sanchez@hfm.tum.de)

Max Engelhardt: orcid.org/0000-0002-9781-7476; email: [engelhardt@hfm.tum.de](mailto:engelhardt@hfm.tum.de)

Appendix SI-A: The derivative method 3

Appendix SI-B: The Gumbel density function 7

Fig. SI-1 Time-sorption isotherms in the L-direction with the DSE fitting curves 13

Fig. SI-2 Time-sorption isotherms in the R-direction with the DSE fitting curves 14

Fig. SI-3 Time-sorption isotherms in the T-direction with the DSE fitting curves 15

Fig. SI-4 Time-sorption isotherms in the L-direction with the RP fitting curves 16

Fig. SI-5 Time-sorption isotherms in the R-direction with the RP fitting curves 17

Fig. SI-6 Time-sorption isotherms in the T-direction with the RP fitting curves 18

Fig. SI-7 Time-sorption isotherms in the L-direction with the SUM fitting curves 19

Fig. SI-8 Time-sorption isotherms in the R-direction with the SUM fitting curves 20

Fig. SI-9 Time-sorption isotherms in the T-direction with the SUM fitting curves 21

Fig. SI-10 Time-sorption isotherms in the L-direction with the DSUM fitting curves 22

Fig. SI-11 Time-sorption isotherms in the R-direction with the DSUM fitting curves 23

Fig. SI-12 Time-sorption isotherms in the T-direction with the DSUM fitting curves 24

Fig. SI-13 Linear correlation D_DSE-1_ *vs*. D_DER_, and β_1_ *vs*. β for the three directions 28

Fig. SI-14 Exponential correlation D_app_ *vs*. Q for the three directions 29

Table SI-1, SI-2 and SI-3 DSE fitting parameters and the corresponding D_DSE_ values 25

Table SI-4 RP fitting parameters and the corresponding D_RP_ values 25

Table SI-5 D_SUM_ values 26

Table SI-6, SI-7 and SI-8 DSUM fitting parameters and the corresponding D_DSUM_ values 26

Table SI-9 t_0.63_ values and the corresponding D_0.63_ values 27

Table SI-10 t_0.5_ values and the corresponding D_0.5_ values 27

Table SI-11 t_max_ values and the corresponding D_DER_ values 27

Table SI-12 FWHM and β values from the DER method 27

Table SI-13 k and $D_{\mathrm{app}}^{\max}$ values for n = 1 for the three directions 30

Table SI-14 k and $D_{\mathrm{app}}^{\max}$ values for n > 1 for the three directions 30

**Appendix SI-A: The derivative method**

In this appendix, the description of this derivative method (DER) for the calculation of the lifetime τ and the shape factor β is explained in detail. A series of simulated curves based on the single-stretched exponential function (SSE) or Weibull equation (Eq. SI-A1) are shown by varying the lifetime τ or the shape factor β to validate the DER method.

$Y=1-e^{-\left( \frac{X}{\tau} \right)^{\beta}}$ (Eq. SI-A1)

Once the SSE curves are simulated (Fig. SI-A1a and SI-A2a) and as already mentioned in the Materials and Methods chapter (see “Derivative method for the determination of the apparent diffusion coefficient”), the steps for the DER procedure are the following:

1) Calculate the logarithm of all X values and plot Y *vs.* log X to obtain the corresponding S-shape curves (Fig. SI-A1b and SI-A2b);

2) Calculate the corresponding derivative dY/d(log X), which results on a peak function (Fig. SI-A1c and SI-A2c);

3) Normalize the dY/d(log X) *vs.* log X curve from 0 to 1 (Fig. SI-A1d and SI-A2d);

4) Localize the log X value corresponding to the maximum in the curve dY/d(log X)_norm_ *vs.* log X;

5) Calculate the FWHM by measuring the distance between the two points, one to the left and one to the right of the local maximum, that satisfy dY/d(log X)_norm_ = 0.5.

In Fig. SI-A1, several SSE curves were simulated by varying the lifetime τ from 50 (red curve) to 150 (blue curve) in 10-unit steps and keeping the shape factor constant (β = 1). After transforming the abscissa values X to the corresponding log X values, all S-shaped curves were derived and normalized, and the corresponding peak function was obtained. The peak maximum is shifted when the τ value varies. Notice that the peak maximum at log t_max_ corresponds to the log τ value; therefore, the lifetime of a set of experimental data can be obtained by calculating the corresponding t_0.63_ – the time at which the Y value is equal to 0.6321 – or by detecting the peak maximum in the derivative curve. After normalizing the derivative curves, the FWHM of each peak remains constant because the shape factor β is constant too.

In Fig. SI-A2, several SSE curves were simulated by varying the shape factor β from 0.6 (red curve) to 1.1 (blue curve) in 0.05-unit steps and keeping the lifetime constant (τ = 100). After transforming the abscissa values X to the corresponding log X values, all S-shaped curves were derived and normalized, and the corresponding peak function was obtained. Notice that the peak maximum of all curves is at log t_max,_ which corresponds to the constant value of log τ = 2. The FWHM values – the distance between the two points at dY/dX_norm_ = 0.5 to the left and to the right of the peak maximum – are inversely proportional to the shape factor, *i.e.*, the smaller the β values, the larger the FWHM, and vice versa.

In order to find out the relationship between the FWHM of the peak function and the shape factor β from the SSE curve, a power law fitting was conducted, showing a reciprocal correlation between the two parameters, *i.e.*, FWHM = 1.06227 β^-1^ (Fig. SI-A3). Therefore, from the analysis of the FWHM in the derivative curve, the shape factor β of the corresponding SSE curve can be evaluated.

| a)   | b)   |
| --- | --- |
| c)   | d)   |

**Fig. SI-A1.** a) Simulated SSE curves varying the lifetime τ from 50 (red curve) to 150 (blue curve) and keeping the shape factor constant (β = 1). b) S-shape simulated SSE curves after transforming the abscissa values from X to log X. c) Derivative curves showing a shift in the peak maximum as a function of the lifetime τ and constant FWHM. d) Normalized derivative curves. Note: the lifetime value τ corresponds to the time at which the Y values equal 1-1/e ≈ 0.6321 = 63.21%.

Finally, in order to verify the independence between the two SSE parameters, *i.e.*, τ and β, three sets of curves were simulated with values of τ = 50, 100 and 150 and varying the shape factor β between 0.6 and 1.1 (Fig. SI-A4). The results show that the peak maximum is directly proportional to the lifetime τ value, and the FWHM is inversely proportional to the shape factor β no matter of the τ value. Therefore, both parameters can be evaluated from the derivative independently.

| a)   | b)   |
| --- | --- |
| c)   | d)   |

**Fig. SI-A2.** a) Simulated SSE curves varying the shape factor β from 0.6 (red curve) to 1.1 (blue curve) and keeping the lifetime t constant (τ = 100). b) S-shape simulated SSE curves after transforming the abscissa values from X to log X. c) Derivative curves showing a change in the peak width as a function of the shape factor β and constant peak maximum log t_max_. d) Normalized derivative curves. Note: the lifetime value τ corresponds to the time at which the Y values equal 1-1/e ≈ 0.6321 = 63.21%.

**Fig. SI-A3.** Reciprocal correlation between the FWHM of the normalized derivatives and the shape factor β values between 0.586 < β < 1.146. Note: data were obtained from Fig. SI-A2d.

| a)   | b)   |
| --- | --- |
| c)   | d)   |

**Fig. SI-A4.** a) Three sets of simulated SSE curves with lifetime t values of 50, 100 and 150 and with shape factor β values from 0.6 (red curve) to 1.1 (blue curve) for each set. b) S-shape simulated SSE curves after transforming the abscissa values from X to log X. c) Derivative curves showing a shift in the peak maximum as a function of the lifetime τ and a change in the peak width as a function of the shape factor β. d) Normalized derivative curves. Note: the lifetime value τ corresponds to the time at which the Y values equal 1-1/e ≈ 0.6321 = 63.21%.

The advantages of this method are that the plateau in the mass uptake during the sorption process does not have to be reached, as soon as the peak maximum in the derivative t_max_ is achieved. Therefore, implementing a numerical protocol following the DER method during the signal monitoring can detect such a t_max_ ≈ τ_1_., and the corresponding apparent diffusion coefficient can be easily calculated. Moreover, from the analysis of the peak’s FWHM, the shape factor β can be calculated, and the diffusion process can be classified, *e.g.*, as a Fickian or non-Fickian process. Finally, there is no need to conduct any fitting procedure or apply any model.

**Appendix SI-B: The Gumbel density function**

In this appendix, the linkage between the single-stretched exponential function (SSE) and the Gumbel density function (GDF) is described. The GDF is a case of the Generalized Extreme Value (GEV) distribution with a peak function or probability density function (PDF) indicated in Eq. SI-B1,

$Y=Ae^{-e^{-\left( \frac{\rho-X}{\omega} \right)}-\frac{\rho-X}{\omega}+1}$ (Eg. SI-B1)

where A is the amplitude, ρ is the peak maximum, and ω is the scale parameter.

A series of single-stretched exponential (SSE) functions was simulated by varying the shape factor β from 0.60 to 1.10 and keeping the lifetime constant τ = 100, which corresponds to the data in Fig. SI-A2b. The DER method was applied in order to obtain the corresponding peak distribution curves and fitted with the GDF (Fig. SI-B1). The fitting results show a perfect correlation between the lifetime τ and the peak value ρ, *i.e.*, τ = 10^ρ^ or ρ = log τ.

| a)   | b)   |
| --- | --- |

**Fig. SI-B1.** Simulated SSE functions (empty symbols) - τ = 100 and 0.60 < β < 1.10 - and the corresponding GDF fitting curves (colored curves) - ρ =2 and 0.72382 < ω < 0.39481. a) 0.60 < β < 0.85 and b) 0.90 < β < 1.10.

Moreover, the mathematical relationship between the shape factor β from the SSE function and the scale parameter ω from the GDF was found to be reciprocal or a power law with an exponent value of n = -1 (Fig. SI-B2), *i.e.*, ω = 0.434295 β^-1^.

**Fig. SI-B2.** Reciprocal correlation between the scale parameter ω (GDF) and the shape factor β (SSE).

Finally, because all DVS data were adjusted with a DSE function, some series of double-stretched exponential (DSSE) functions were simulated by keeping constant the fast sorption process (τ_1_ = 10) and varying the slow sorption process (50 < τ_2_ < 1000) with different shape factor values β_1_ and β_2_. As before, the DER method was applied, the corresponding peak distribution curves were obtained and fitted with a double-Gumbel density function (DGDF) (Fig. SI-B3, SI-B4 and SI-B5). In this way, the effect on the τ_2_, β_1_ and β_2_ values on the peak maximum t_max_ was evaluated, and the shift percentage with respect to the τ_1_ was calculated for each case (Fig. SI-B6). The results show that the maximum shift between the peak maximum in the total curve t_max_, and the peak maximum of the fast sorption process τ_1_ is below 10%. Therefore, when the lifetime of the slow sorption process is far larger than that of the fast sorption process, one can assume t_max_ ≈ τ_1_ by only localizing the peak maximum from the experimental DVS data.

| a)   | b)   |
| --- | --- |
| c)   | d)   |
| e)   | f)   |

**Fig. SI-B3.** Series of simulated DSE curves (empty symbols) - τ_1_ = 10, β_1_ = 1.0, β_2_ = 1.0, and τ_2_ = a) 50, b) 100, c) 250, d) 500, and e) 1000 - and the corresponding double GDF (DGDF) fitting curves (red). F) DGDF fitting curves for the different τ_2_ values, *i.e*., 50, 100, 250, 500 and 1000.

| a)   | b)   |
| --- | --- |
| c)   | d)   |
| e)   | f)   |

**Fig. SI-B4.** Series of simulated DSE curves (empty symbols) - τ_1_ = 10, β_1_ = 0.6, β_2_ = 0.6, and τ_2_ = a) 50, b) 100, c) 250, d) 500, and e) 1000 - and the corresponding double GDF (DGDF) fitting curves (red). F) DGDF fitting curves for the different τ_2_ values, *i.e*., 50, 100, 250, 500 and 1000.

| a)   | b)   |
| --- | --- |
| c)   | d)   |
| e)   | f)   |

**Fig. SI-B5.** Series of simulated DSE curves (empty symbols) - τ_1_ = 10, β_1_ = 1.0, β_2_ = 0.6, and τ_2_ = a) 50, b) 100, c) 250, d) 500, and e) 1000 - and the corresponding double GDF (DGDF) fitting curves (red). F) DGDF fitting curves for the different τ_2_ values, *i.e*., 50, 100, 250, 500 and 1000.

**Fig. SI-B6.** Peak maximum t_max_ - from the fitting of the simulated DSE function with a DGDF - as a function of the slow process lifetime τ_2_ with the constant value τ_1_ = 10, and for different shape factor values β_1_ and β_2_.

| a)   |  |
| --- | --- |
| b)   |  |
| c)   |  |
| d)   |  |
| e)   |  |

**Fig. SI-1.** lin-lin (left column) and lin-log (right column) plots of the time-sorption isotherm DVS data at different flow rate values Q for the spruce disk in the **L-direction**, together with the corresponding **DSE** fitting curve.

| a)   |  |
| --- | --- |
| b)   |  |
| c)   |  |
| d)   |  |
| e)   |  |

**Fig. SI-2.** lin-lin (left column) and lin-log (right column) plots of the time-sorption isotherm DVS data at different flow rate values Q for the spruce disk in the **R-direction**, together with the corresponding **DSE** fitting curve.

| a)   |  |
| --- | --- |
| b)   |  |
| c)   |  |
| d)   |  |
| e)   |  |

**Fig. SI-3.** lin-lin (left column) and lin-log (right column) plots of the time-sorption isotherm DVS data at different flow rate values Q for the spruce disk in the **T-direction**, together with the corresponding **DSE** fitting curve.

| a)   |  |
| --- | --- |
| b)   |  |
| c)   |  |
| d)   |  |

**Fig. SI-4.** lin-lin (left column) and lin-log (right column) plots of the time-sorption isotherm DVS data at different flow rate values Q for the spruce disk in the **L-direction**, together with the corresponding **RP** fitting curve.

| a)   |  |
| --- | --- |
| b)   |  |
| c)   |  |
| d)   |  |

**Fig. SI-5.** lin-lin (left column) and lin-log (right column) plots of the time-sorption isotherm DVS data at different flow rate values Q for the spruce disk in the **R-direction**, together with the corresponding **RP** fitting curve.

| a)   |  |
| --- | --- |
| b)   |  |
| c)   |  |
| d)   |  |

**Fig. SI-6.** lin-lin (left column) and lin-log (right column) plots of the time-sorption isotherm DVS data at different flow rate values Q for the spruce disk in the **T-direction**, together with the corresponding **RP** fitting curve.

| a)   |  |
| --- | --- |
| b)   |  |
| c)   |  |
| d)   |  |
| e)   |  |

**Fig. SI-7.** lin-lin (left column) and lin-log (right column) plots of the time-sorption isotherm DVS data at different flow rate values Q for the spruce disk in the **L-direction**, together with the corresponding **SUM** fitting curve.

| a)   |  |
| --- | --- |
| b) |  |
| c) |  |
| d) |  |
| e) |  |

**Fig. SI-8.** lin-lin (left column) and lin-log (right column) plots of the time-sorption isotherm DVS data at different flow rate values Q for the spruce disk in the **R-direction**, together with the corresponding **SUM** fitting curve.

| a) |  |
| --- | --- |
| b) |  |
| c) |  |
| d) |  |
| e) |  |

**Fig. SI-9.** lin-lin (left column) and lin-log (right column) plots of the time-sorption isotherm DVS data at different flow rate values Q for the spruce disk in the **T-direction**, together with the corresponding **SUM** fitting curve.

| a) |  |
| --- | --- |
| b) |  |
| c) |  |
| d) |  |
| e) |  |

**Fig. SI-10.** lin-lin (left column) and lin-log (right column) plots of the time-sorption isotherm DVS data at different flow rate values Q for the spruce disk in the **L-direction**, together with the corresponding **DSUM** fitting curve.

| a) |  |
| --- | --- |
| b) |  |
| c) |  |
| d) |  |
| e) |  |

**Fig. SI-11.** lin-lin (left column) and lin-log (right column) plots of the time-sorption isotherm DVS data at different flow rate values Q for the spruce disk in the **R-direction**, together with the corresponding **DSUM** fitting curve.

| a) |  |
| --- | --- |
| b) |  |
| c) |  |
| d) |  |
| e) |  |

**Fig. SI-12.** lin-lin (left column) and lin-log (right column) plots of the time-sorption isotherm DVS data at different flow rate values Q for the spruce disk in the **T-direction**, together with the corresponding **DSUM** fitting curve.

**Table SI-1.** Time-sorption isotherm DSE fitting parameters and the apparent diffusion coefficient D_DSE_ at the different flow rate values Q for the spruce disk in the L-direction.

| Q (cm^3^/min) | A_1_ | A_2_ | τ_1_ (min) | τ_2_ (min) | β_1_ | β_2_ | τ (min) | β | D_DSE_ (10^-10^ m^2^/s) |
| --- | --- | --- | --- | --- | --- | --- | --- | --- | --- |
| 10 | 0.46±0.01 | 0.54±0.01 | 6.3±0.2 | 905±11 | 1.1±0.1 | 0.59±0.01 | 340±4 | 0.586±0.003 | 0.098±0.001 |
| 50 | 0.813±0.004 | 0.187±0.004 | 53.7±0.1 | 209±8 | 1.086±0.003 | 0.59±0.01 | 61±1 | 0.720±0.004 | 0.55±0.01 |
| 100 | 0.869±0.003 | 0.131±0.003 | 24.2±0.1 | 218±9 | 0.982±0.003 | 0.59±0.01 | 27.7±0.3 | 0.651±0.004 | 1.20±0.01 |
| 150 | 0.916±0.002 | 0.084±0.002 | 20.48±0.03 | 210±8 | 0.992±0.002 | 0.59±0.01 | 22.3±0.2 | 0.78±0.01 | 1.49±0.01 |
| 200 | 0.933±0.002 | 0.067±0.002 | 18.55±0.03 | 195±10 | 0.993±0.002 | 0.59±0.01 | 19.8±0.2 | 0.82±0.01 | 1.68±0.01 |

**Table SI-2.** Time-sorption isotherm DSE fitting parameters and the apparent diffusion coefficient D_DSE_ at the different flow rate values Q for the spruce disk in the R-direction.

| Q (cm^3^/min) | A_1_ | A_2_ | τ_1_ (min) | τ_2_ (min) | β_1_ | β_2_ | τ (min) | β | D_DSE_ (10^-10^ m^2^/s) |
| --- | --- | --- | --- | --- | --- | --- | --- | --- | --- |
| 10 | 0.87±0.01 | 0.13±0.01 | 37.4±0.3 | 611±18 | 0.85±0.01 | 1.15±0.03 | 51±1 | 0.586±0.003 | 0.65±0.01 |
| 50 | 0.821±0.002 | 0.179±0.002 | 79.7±0.1 | 260±6 | 1.046±0.002 | 0.60±0.01 | 89±1 | 0.760±0.004 | 0.371±0.003 |
| 100 | 0.889±0.002 | 0.111±0.002 | 43.41±0.04 | 289±9 | 1.004±0.002 | 0.63±0.01 | 48.2±0.4 | 0.756±0.004 | 0.69±0.01 |
| 150 | 0.910±0.003 | 0.090±0.003 | 39.33±0.04 | 230±12 | 0.985±0.002 | 0.60±0.01 | 42.5±0.3 | 0.80±0.01 | 0.78±0.01 |
| 200 | 0.912±0.003 | 0.088±0.003 | 36.49±0.04 | 210±13 | 0.976±0.002 | 0.59±0.01 | 39.3±0.3 | 0.80±0.01 | 0.84±0.01 |

**Table SI-3.** Time-sorption isotherm DSE fitting parameters and the apparent diffusion coefficient D_DSE_ at the different flow rate values Q for the spruce disk in the T-direction

| Q (cm^3^/min) | A_1_ | A_2_ | τ_1_ (min) | τ_2_ (min) | β_1_ | β_2_ | τ (min) | β | D_DSE_ (10^-10^ m^2^/s) |
| --- | --- | --- | --- | --- | --- | --- | --- | --- | --- |
| 10 | 0.872±0.002 | 0.128±0.002 | 45.9±0.2 | 1106±11 | 0.779±0.003 | 1.15±0.01 | 66±1 | 0.586±0.003 | 0.57±0.01 |
| 50 | 0.810±0.002 | 0.190±0.002 | 81.1±0.1 | 228±5 | 1.097±0.002 | 0.61±0.01 | 90±1 | 0.795±0.005 | 0.426±0.003 |
| 100 | 0.883±0.002 | 0.117±0.002 | 44.31±0.03 | 290±9 | 1.042±0.002 | 0.59±0.01 | 49.1±0.4 | 0.768±0.004 | 0.78±0.01 |
| 150 | 0.903±0.002 | 0.097±0.002 | 41.28±0.03 | 291±9 | 1.010±0.001 | 0.59±0.01 | 43.4±0.4 | 0.80±0.01 | 0.88±0.01 |
| 200 | 0.911±0.002 | 0.089±0.002 | 38.75±0.02 | 200±7 | 1.006±0.001 | 0.64±0.01 | 41.7±0.3 | 0.83±0.01 | 0.91±0.01 |

**Table SI-4.** Time-sorption isotherm RP fitting parameters and the apparent diffusion coefficient D_RP_ at the different flow rate values Q for the spruce disk in the L-, R- and T-direction.

| Q (cm^3^/min) | τ (L) (min) | τ (R) (min) | τ (T) (min) | n (L) | n (R) | n (T) | D_RP_ (L) (10^-10^ m^2^/s) | D_RP_ (R) (10^-10^ m^2^/s) | D_RP_ (T) (10^-10^ m^2^/s) |
| --- | --- | --- | --- | --- | --- | --- | --- | --- | --- |
| 50 | 100±0 | 145±1 | 143±1 | 0.857±0.001 | 0.842±0.005 | 0.867±0.005 | 0.333±0.001 | 0.227±0.002 | 0.267±0.002 |
| 100 | 37±1 | 66±1 | 67±1 | 0.916±0.011 | 0.908±0.012 | 0.922±0.011 | 0.893±0.015 | 0.505±0.009 | 0.569±0.009 |
| 150 | 31±1 | 60±1 | 60±1 | 0.914±0.012 | 0.881±0.012 | 0.880±0.012 | 1.078±0.020 | 0.554±0.011 | 0.632±0.012 |
| 200 | 27±1 | 54±1 | 59±1 | 0.925±0.015 | 0.885±0.014 | 0.883±0.012 | 1.236±0.026 | 0.615±0.014 | 0.651±0.013 |

**Table SI-5.** The apparent diffusion coefficient D_SUM_ at the different flow rate values Q for the spruce disk in the L-, R and T-direction.

| Q (cm^3^/min) | D_SUM_ (L) (10^-10^ m^2^/s) | D_SUM_ (R) (10^-10^ m^2^/s) | D_SUM_ (T) (10^-10^ m^2^/s) |
| --- | --- | --- | --- |
| 10 | 0.094 | 0.232 | 0.193 |
| 50 | 0.217 | 0.151 | 0.151 |
| 100 | 0.459 | 0.275 | 0.273 |
| 150 | 0.593 | 0.316 | 0.302 |
| 200 | 0.676 | 0.341 | 0.323 |

**Table SI-6.** Time-sorption isotherm DSUM fitting parameters and the apparent diffusion coefficient D_DSUM_ at the different flow rate values Q for the spruce disk in the L-direction.

| Q (cm^3^/min) | A_1_ | A_2_ | D_1_ (10^-10^ m^2^/s) | D_2_ (10^-10^ m^2^/s) | D_DSUM_ (10^-10^ m^2^/s) |
| --- | --- | --- | --- | --- | --- |
| 10 | 0.534 | 0.466 | 1.87 | 0.019 | 0.059 |
| 50 | 0.929 | 0.071 | 0.248 | 0.021 | 0.225 |
| 100 | 0.922 | 0.078 | 0.555 | 0.033 | 0.495 |
| 150 | 0.952 | 0.048 | 0.665 | 0.032 | 0.621 |
| 200 | 0.963 | 0.037 | 0.738 | 0.033 | 0.702 |

**Table SI-7.** Time-sorption isotherm DSUM fitting parameters and the apparent diffusion coefficient D_DSUM_ at the different flow rate values Q for the spruce disk in the R-direction.

| Q (cm^3^/min) | A_1_ | A_2_ | D_1_ (10^-10^ m^2^/s) | D_2_ (10^-10^ m^2^/s) | D_DSUM_ (10^-10^ m^2^/s) |
| --- | --- | --- | --- | --- | --- |
| 10 | 0.818 | 0.182 | 0.362 | 0.031 | 0.255 |
| 50 | 0.940 | 0.060 | 0.166 | 0.013 | 0.152 |
| 100 | 0.943 | 0.057 | 0.309 | 0.022 | 0.285 |
| 150 | 0.959 | 0.041 | 0.343 | 0.023 | 0.325 |
| 200 | 0.961 | 0.039 | 0.370 | 0.025 | 0.351 |

**Table SI-8.** Time-sorption isotherm DSUM fitting parameters and the apparent diffusion coefficient D_DSUM_ at the different flow rate values Q for the spruce disk in the T-direction

| Q (cm^3^/min) | A_1_ | A_2_ | D_1_ (10^-10^ m^2^/s) | D_2_ (10^-10^ m^2^/s) | D_DSUM_ (10^-10^ m^2^/s) |
| --- | --- | --- | --- | --- | --- |
| 10 | 0.815 | 0.185 | 0.297 | 0.018 | 0.198 |
| 50 | 0.948 | 0.052 | 0.163 | 0.011 | 0.152 |
| 100 | 0.944 | 0.056 | 0.305 | 0.019 | 0.282 |
| 150 | 0.956 | 0.044 | 0.329 | 0.020 | 0.310 |
| 200 | 0.965 | 0.035 | 0.347 | 0.025 | 0.331 |

**Table SI-9.** Apparent diffusion coefficient D_0.63_ calculated from the lifetime value t_0.63_ at the different flow rate values Q for the spruce disk in the L-, R- and T-direction.

| Q (cm^3^/min) | t_0.63_ (L) (min) | t_0.63_ (R) (min) | t_0.63_ (T) (min) | D_0.63_ (L) (10^-10^ m^2^/s) | D_0.63_ (R) (10^-10^ m^2^/s) | D_0.63_ (T) (10^-10^ m^2^/s) |
| --- | --- | --- | --- | --- | --- | --- |
| 50 | 64 | 98 | 101 | 1.59 | 0.814 | 0.776 |
| 100 | 30 | 52 | 52 | 1.39 | 0.726 | 0.726 |
| 150 | 24 | 46 | 46 | 1.11 | 0.642 | 0.642 |
| 200 | 21 | 41 | 43 | 0.522 | 0.341 | 0.331 |

**Table SI-10.** Apparent diffusion coefficient D_0.5_ calculated from the half-life time value t_0.5_ at the different flow rate values Q for the spruce disk in the L-, R- and T-direction.

| Q (cm^3^/min) | t_0.5_ (L) (min) | t_0.5_ (R) (min) | t_0.5_ (T) (min) | D_0.5_ (L) (10^-10^ m^2^/s) | D_0.5_ (R) (10^-10^ m^2^/s) | D_0.5_ (T) (10^-10^ m^2^/s) |
| --- | --- | --- | --- | --- | --- | --- |
| 50 | 44 | 67 | 71 | 0.189 | 0.124 | 0.117 |
| 100 | 20 | 36 | 36 | 0.417 | 0.231 | 0.231 |
| 150 | 17 | 32 | 32 | 0.490 | 0.260 | 0.260 |
| 200 | 15 | 28 | 30 | 0.555 | 0.298 | 0.278 |

**Table SI-11.** Apparent diffusion coefficient D_app_ calculated from the time at the local maximum t_max_ from the DER method at the different flow rate values Q for the spruce disk in the L-, R- and T-direction.

| Q (cm^3^/min) | t_max_ (L) (min) | t_max_ (R) (min) | t_max_ (T) (min) | D_DER_ (L) (10^-10^ m^2^/s) | D_DER_ (R) (10^-10^ m^2^/s) | D_DER_ (T) (10^-10^ m^2^/s) |
| --- | --- | --- | --- | --- | --- | --- |
| 50 | 52.5 | 79.7 | 82.1 | 0.636 | 0.415 | 0.465 |
| 100 | 20.8 | 40.0 | 43.3 | 1.603 | 0.826 | 0.882 |
| 150 | 18.2 | 37.0 | 39.5 | 1.830 | 0.894 | 0.967 |
| 200 | 16.4 | 34.4 | 37.8 | 2.041 | 0.961 | 1.009 |

**Table SI-12.** Shape factor β calculated from the peak’s FWHM from the DER method at the different flow rate values Q for the spruce disk in the L-, R- and T-direction.

| Q (cm^3^/min) | FWHM (L) | FWHM (R) | FWHM | β (L) | β (R) | β (T) |
| --- | --- | --- | --- | --- | --- | --- |
| 50 | 0.926 | 1.055 | 1.007 | 1.147 | 1.007 | 1.054 |
| 100 | 1.024 | 1.095 | 1.057 | 1.038 | 0.970 | 1.005 |
| 150 | 1.017 | 1.123 | 1.101 | 1.045 | 0.946 | 0.965 |
| 200 | 1.036 | 1.131 | 1.109 | 1.025 | 0.939 | 0.958 |

| a) |
| --- |
| b) |

**Fig. SI-13.** a) Linear relationship between the apparent diffusion coefficient D_DSE-1_ from the DSE fitting model and the shape factor β from the DER method for the three wood directions, *i.e.*, L, R and T, and flow rate Q values. b) Linear relationship between the shape factor β_1_ from the DSE fitting model and the shape factor β from the DER method for the three wood directions, *i.e.*, L, R and T, and flow rate Q values. Note: 95% confidence band (deep green), 95% prediction band (light green)

| a) | b) |
| --- | --- |
| c) | d) |
| e) | f) |
| g) | h) |

**Fig. SI-14.** Apparent diffusion coefficient (D_app_) values from 30% to 80% RH as a function of the flow rate (Q) for the three wood directions, *i.e.*, L, R and T, obtained from of the experimental data in Figure 1 using the different fitting models and approaches, *i.e.*, a) the derivative method (DER), b) the double-stretched exponential function (DSE), c) the fast sorption process in the double-stretched exponential function (DSE-1), c) the lifetime value (t_0.63_), d) the Ritger-Peppas function (RP), e) the Fickian function (SUM), f) the double-Fickian function (DSUM), and g) the half-life time value (t_0.5_). The curves correspond to the data in the three wood directions, following an exponential function (n = 1, dashed lines) and a stretched exponential function (n > 1, solid lines).

**Table SI-13.** Flow rate constant k and maximum apparent diffusion coefficient $D_{\mathrm{app}}^{\max}$ from the fitting (n = 1) of the apparent diffusion coefficient D_app_ at different flow rate values Q obtained from the derivative method (DER), the double-stretched exponential function (DSE), the fast sorption process in the double-stretched exponential function (DSE-1), the lifetime value (t_0.63_), the Ritger-Peppas function (RP), the Fickian function (SUM), the double-Fickian function (DSUM), and the half-life time value (t_0.5_) for the spruce disk in the L-, R- and T-direction.

|  | L | | R | | T | |
| --- | --- | --- | --- | --- | --- | --- |
|  | k (10^-3^ min/cm^3^) | $D_{\mathrm{app}}^{\max}$(10^-10^ cm^3^/min | k (10^-3^ min/cm^3^) | $D_{\mathrm{app}}^{\max}$(10^-10^ cm^3^/min | k (10^-3^ min/cm^3^) | $D_{\mathrm{app}}^{\max}$(10^-10^ cm^3^/min |
| DER | 13±2 | 2.18±0.04 | 19±3 | 0.97±0.03 | 20±1 | 1.03±0.01 |
| DSE | 9.2±0.1 | 2.00±0.01 | 15±1 | 0.88±0.02 | 17±1 | 0.95±0.01 |
| DSE-1 | 12±1 | 1.97±0.04 | 12±2 | 0.93±0.03 | 21±8 | 0.97±0.04 |
| t_0.63_ | 8.5±0.3 | 1.94±0.03 | 13±2 | 0.86±0.04 | 16±1 | 0.81±0.01 |
| RP | 10±1 | 1.44±0.04 | 15±3 | 0.63±0.04 | 19±1 | 0.67±0.01 |
| SUM | 7.5±0.3 | 0.87±0.02 | 14±1 | 0.36±0.01 | 17±2 | 0.33±0.01 |
| DSUM | 8.7±0.1 | 0.85±0.00 | 15±1 | 0.37±0.01 | 18±2 | 0.34±0.01 |
| t_0.5_ | 11±1 | 0.62±0.03 | 13±3 | 0.32±0.03 | 16±1 | 0.29±0.01 |

**Table SI-14.** Flow rate constant k and maximum apparent diffusion coefficient $D_{\mathrm{app}}^{\max}$ from the fitting (n > 1) of the apparent diffusion coefficient D_app_ at different flow rate values Q obtained from the derivative method (DER), the double-stretched exponential function (DSE), the fast sorption process in the double-stretched exponential function (DSE-1), the lifetime value (t_0.63_), the Ritger-Peppas function (RP), the Fickian function (SUM), the double-Fickian function (DSUM), and the half-life time value (t_0.5_) for the spruce disk in the L-, R- and T-direction.

|  | L | | R | | T | |
| --- | --- | --- | --- | --- | --- | --- |
|  | k (10^-3^ min/cm^3^) | $D_{\mathrm{app}}^{\max}$(10^-10^ cm^3^/min | k (10^-3^ min/cm^3^) | $D_{\mathrm{app}}^{\max}$(10^-10^ cm^3^/min | k (10^-3^ min/cm^3^) | $D_{\mathrm{app}}^{\max}$(10^-10^ cm^3^/min |
| DER | 12.5±0.4 | 1.98±0.04 | 14.9±0.4 | 0.94±0.02 | 15.4±0.3 | 1.00±0.01 |
| DSE | 11.0±0.4 | 1.71±0.04 | 14.0±0.4 | 0.84±0.02 | 14.9±0.2 | 0.91±0.01 |
| DSE-1 | 12.3±0.4 | 1.78±0.04 | 12.3±0.4 | 0.89±0.02 | 16.2±0.4 | 0.94±0.03 |
| t_0.63_ | 10.5±0.4 | 1.65±0.04 | 13.1±0.4 | 0.81±0.03 | 14.1±0.4 | 0.77±0.01 |
| RP | 11.2±0.4 | 1.21±0.04 | 13.6±0.4 | 0.59±0.02 | 14.7±0.2 | 0.64±0.01 |
| SUM | 10.0±0.2 | 0.72±0.01 | 13.8±0.4 | 0.34±0.01 | 15.0±0.4 | 0.32±0.01 |
| DSUM | 10.8±0.3 | 0.72±0.01 | 13.8±0.4 | 0.35±0.01 | 15.0±0.4 | 0.33±0.01 |
| t_0.5_ | 11.8±0.4 | 0.55±0.02 | 12.7±0.4 | 0.30±0.01 | 14.2±0.4 | 0.27±0.01 |
